# Vinpocetine—An “Old” Drug with a New Face: Moving Toward a Better Understanding of Its Neuroprotective Mechanism of Action

**DOI:** 10.3390/biom16030454

**Published:** 2026-03-17

**Authors:** E. Sylvester Vizi, Béla Kiss

**Affiliations:** 1 HUN-REN Institute of Experimental Medicine, Szigony-u 43, H-1083 Budapest, Hungary; 2 Department of Pharmacology and Pharmacotherapy, Semmelweis University, Nagyvárad tér 4, H-1085 Budapest, Hungary; 3 Gedeon Richter Plc, Gyömrői u, 19-21, H-1103 Budapest, Hungary; bkiss46@gmail.com

**Keywords:** vinpocetine, cerebral ischemia, PDE1, sodium channels, neuroinflammation, oxidative stress, neuroprotection

## Abstract

Synthesized more than 60 years ago, vinpocetine—the active ingredient of Cavinton^®^, with over five decades of clinical use—has remained the subject of extensive investigation, particularly during the past 15 years. During this time, a large body of experimental preclinical evidence has accumulated demonstrating its neuroprotective potential and complex mechanisms of action in cerebral ischemia–hypoxia. Comprehensive in vitro studies and animal experiments have significantly elucidated the molecular basis of vinpocetine and the signaling pathways through which it prevents or mitigates ischemic injury. In this review, we summarize earlier and more recent experimental results that highlight the multifaceted nature of vinpocetine’s neuroprotective actions, which include inhibition of phosphodiesterase type 1, blockade of voltage-dependent NaV1.8 channels, reduction of oxidative stress, and suppression of neuroinflammatory processes triggered by cerebral ischemia–hypoxia. Taken together, it can be hypothesized that, under in vivo conditions, vinpocetine’s individual actions are additive or synergistic, thereby contributing in a combined manner to recovery from cerebral ischemic insult.

## 1. Introduction

This review aims to summarize in vivo and in vitro experimental findings related to vinpocetine’s primary indications—including cerebral ischemia, ischemia–reperfusion, cerebral hypoperfusion, and their consequences—with the goal of clarifying its molecular mechanism of action. It should be noted that, in addition to studies on vinpocetine’s effects in cerebral ischemia and hypoxia, nearly 100 further publications describe its protective actions against various toxic agents (e.g., manganese, lead, toluene, carbon tetrachloride, ethanol) or drug-induced adverse effects (e.g., doxorubicin, methotrexate, chloroquine). Moreover, beneficial effects have been reported in animal models of diverse conditions (e.g., in animal model of diseases such as depression, inflammatory diseases, β-amyloid-related cerebral changes, cognitive deficits after brain trauma, Parkinsonism, schizophrenia) affecting organs such as the brain, liver, heart, lungs, and the vascular system. These findings illustrate the remarkably broad pharmacological profile of vinpocetine and contribute to a better understanding of its mechanism of action.

Recent reviews addressing vinpocetine’s actions in brain ischemia–hypoxia and in various non-ischemic animal disease models are summarized in Table 1. These reviews and experimental results reviewed here clearly demonstrate continuing considerable scientific interest toward vinpocetine.

## 2. Vinpocetine

**Figure 1:**
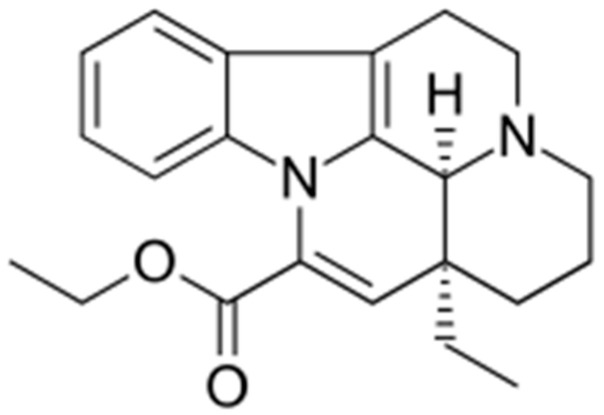
Structure of vinpocetine ([(3α,16α)-eburnamenine-14-carboxylic acid ethyl ester]) (Mw: 350.5).

Vinpocetine (Figure 1), a semisynthetic derivative of vincamine—the *Vinca minor* alkaloid—was synthesized in Hungary in the mid-1960s. The first publications describing its pharmacology and clinical effects appeared in the mid-1970s [29]. It was marketed under the brand name Cavinton^®^ in Hungary for the treatment of cerebrovascular disorders, stroke, post-stroke conditions, and vascular dementia. The total synthesis of vinpocetine was published in 1983 [30].

## 3. Early Pharmacological Studies

Vinpocetine was shown to enhance carotid blood flow and reduce vascular resistance in dogs [31]. Early pharmacological investigations demonstrated the antihypoxic and anti-ischemic properties of vinpocetine. Initially, the protective or ameliorating effects of vinpocetine against symptoms of cerebral hypoxia, ischemia, and ischemia–reperfusion injury were primarily attributed to its ability to enhance cerebral blood flow in various animal models [32,33,34,35,36,37,38,39,40].

Antihypoxic effects of vinpocetine were also demonstrated in primary hippocampal cultures [41]. Its anti-ischemic and antihypoxic efficacy was further confirmed by reports showing increased local blood flow and glucose utilization in the CA1 region of the rat hippocampus under post-ischemic conditions [42]. Increased cerebral blood flow and improved glucose utilization were also demonstrated in human studies [43]. In rats, vinpocetine pretreatment prevented ischemia-induced damage in peripheral organs, such as the retina [44], kidney [45], and liver [46,47].

Over the 60 years since vinpocetine’s clinical introduction, scientific interest has remained constant. Numerous novel actions have been discovered in laboratory studies, and several new derivatives have been synthesized to improve pharmacodynamic efficacy and pharmacokinetic properties [48,49,50].

## 4. Molecular Targets of Vinpocetine

Vinpocetine belongs to the group of therapeutics that—based on the in vitro and in vivo data available so far—exert their pharmacological actions via multiple molecular targets. Vinpocetine’s known molecular targets are summarized in Table 2.

Early radioligand binding assays indicated that vinpocetine does not display affinity (IC_50_ >> 1–10 µM) for rat cortical adrenergic α_1_, α_2_, β_1_, β_2_, dopamine D4.2, adenosine A_1_, serotonin 5-HT_2_, hippocampal 5-HT_1_, striatal dopamine D_2_ receptors, nor for monoamine uptake sites or cytochrome P450 enzymes CYP2D6 and CYP3A4 [59,62,63].

## 5. Neuroprotective Effects of Vinpocetine

**Figure 2:**
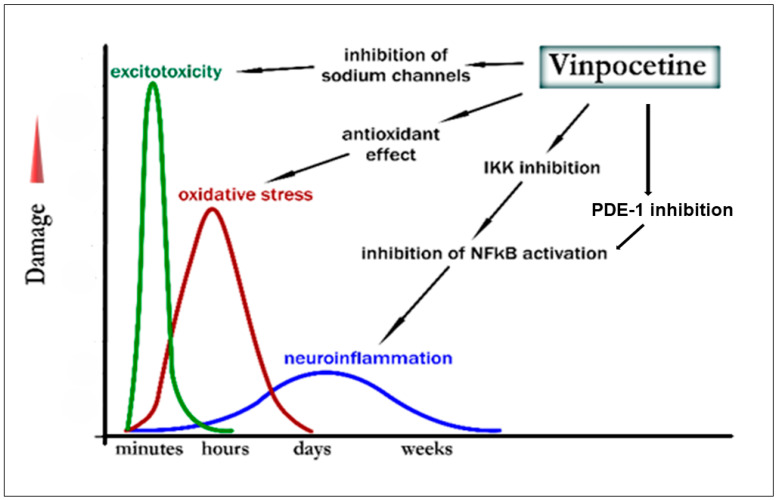
Effects of vinpocetine on excitotoxicity, oxidative stress, and neuroinflammation in their order of appearance following ischemia (adapted for vinpocetine after [68]).

During cerebral ischemia or stroke—conditions involving local or global interruption of glucose and oxygen supply—several pathological molecular events occur rapidly and sequentially: ATP depletion, impaired Na^+^/K^+^-ATPase activity, neuronal depolarization, intracellular Ca^2+^ accumulation, and uncontrolled release of neurotransmitters such as the excitatory glutamate. Excessive glutamate leads to overactivation of NMDA receptors, ultimately resulting in neuronal damage or death via excitotoxicity and related processes, including oxidative stress, mitochondrial dysfunction, and neuroinflammation, which contribute to the progression of ischemic injury [64,65,66,67]. The ischemia-induced sequential appearance of the damaging events along with the potential effects of vinpocetine thereon is depicted in Figure 2.

### 5.1. Vinpocetine Inhibits Glutamate Excitotoxicity

In cerebrocortical cell cultures, vinpocetine inhibited glutamate- and glutamate agonist-induced cytotoxicity (NMDA, quisqualate), as assessed by LDH release [69]. In striatal slices, it inhibited dopamine and acetylcholine release induced by NMDA, quisqualate, and AMPA, but not kainate. In vitro binding experiments suggested a possible interaction of vinpocetine with quisqualate/AMPA receptors [70]. Vinpocetine also inhibited [^3^H]glutamate release from hippocampal synaptosomes induced by veratridine or 4-amino-pyridine (4-AP) [71,72].

In Xenopus oocytes expressing NMDA receptors, vinpocetine inhibited NMDA responses but did not affect AMPA or kainate responses; it slightly inhibited [^3^H]MK-801 binding (IC_50_ = 110 µM). These data suggested that vinpocetine might close the NMDA receptor channel gate similarly to Zn^2+^ [60].

The protective effect of vinpocetine against NMDA-induced neuronal injury was also demonstrated in vivo. Microinjection of NMDA (30 nM) into the rat entorhinal cortex induced behavioral and cognitive impairments (novel object recognition, social discrimination, spontaneous alternation, spatial learning in the Morris water maze). These deficits were prevented entirely by vinpocetine (10 mg/kg, i.p., administered at 60 and 90 min after NMDA injection, then twice daily for three days) [73].

### 5.2. Vinpocetine Inhibits Voltage-Dependent Na^+^-Channels (Na_v_)

The polarization of the cell membrane is determined by the difference between intra- and extracellular Na^+^ concentrations. Na^+^ influx occurs through specialized voltage-gated sodium channels expressed in skeletal muscle, cardiac muscle, and neuronal membranes. In nerve terminals, Na^+^ influx plays a pivotal role: when followed by Ca^2+^ entry, it triggers vesicular transmitter release and thereby mediates synaptic communication.

Numerous clinically used drugs safely modulate sodium channel function, including local anesthetics, anticonvulsants, and antiarrhythmics [74]. Na^+^-channel blockers are used to treat neuropathic pain, muscle spasm, Alzheimer’s disease, and amyotrophic lateral sclerosis (ALS) [75,76]. Significantly, these agents typically do not fully inhibit channel activity but instead modulate channel kinetics—most commonly by slowing inactivation-gate reopening.

A substantial proportion of approved drugs exhibit some degree of sodium-channel modulation. One study reported that 25% of therapeutic agents inhibit sodium channels [77], while another demonstrated that most of 656 drugs tested across 73 molecular targets showed measurable activity on Na^+^ channels [78]. Notably, these findings were obtained at relatively high concentrations (10–30 μM), which are far above therapeutic plasma levels. When Na^+^-channel effects occur at clinically relevant concentrations, further investigation is required to determine whether these effects mediate therapeutic benefits or represent side effects. For example, the selective dopamine-uptake inhibitor GBR 12909 (vanorexine) is an effective Na^+^-channel blocker at low micromolar concentrations [79]. Vanorexine was subsequently found to have antiarrhythmic properties, and it progressed to phase 2 trials [80,81].

Many antidepressants and antipsychotics are also potent sodium-channel modulators. In a comparative study of 44 therapeutic drugs, 14 exhibited Na^+^-channel-blocking activity, and nine were active at therapeutic concentrations. Four of five antipsychotics tested were effective inhibitors, three of which acted within physiological concentration ranges [74]. The reason Na^+^-channel inhibition is common among these therapeutic classes remains unclear.

For neuroprotective drugs (vinpocetine also belongs to this class), the phenomenon is more easily explained. Acute and chronic neurodegeneration is frequently accompanied by neuronal hyperexcitability, which can be attenuated by fine-tuned Na^+^-channel modulation. Importantly, the therapeutic aim is not maximal inhibition but selective damping of pathological hyperactivity in damaged or compromised neuronal populations. Such dysfunction may arise from trauma, ischemia, or inflammation, conditions under which neurons fail to maintain membrane potential and become prone to uncontrolled firing, a process implicated in epilepsy, neuropathic pain, arrhythmias, and spasticity.

Na^+^-channel modulators display marked state dependence: affinity varies with the channel’s conformational state. The three principal states are resting (activation gate closed), open (both gates open), and inactivated (inactivation gate closed). The latter two states are depolarization-dependent, and sodium-channel inhibitors typically show orders-of-magnitude higher affinity for these conformations. Because damaged neurons are more depolarized and fire more frequently, Na^+^-channel blockers preferentially target precisely those cells contributing to pathological activity. Thus, modulation is typically superior to complete blockade.

This raises the question of whether ion-conductance blockade can be dissociated from channel modulation. Although pore-binding would theoretically inhibit both processes, experimental and structural findings demonstrate that such dissociation is possible. For example, the neuroprotective and anticonvulsant drug riluzole can modulate Na^+^-channels without directly blocking the pore. Cryo-electron microscopy and other structural methods show that riluzole binds within fenestrations—side chambers open toward the lipid bilayer—rather than within the conduction pore [82,83]. Voltage-gated Na^+^-channels possess not only two functional gates but also four fenestrations. These do not conduct Na^+^ ions, as they connect to the lipid bilayer rather than the aqueous compartments, but they allow membrane-soluble compounds to access intrachannel binding sites. Structural studies using cryo-EM [84], X-ray crystallography, NMR spectroscopy [85], and molecular dynamics simulations [86] confirm that fenestrations serve as key drug-binding sites and that riluzole’s inhibitory action is primarily modulatory. Recent advances in cryo-EM have accelerated the structural elucidation of membrane proteins and their drug-binding sites.

Early work demonstrated that Na^+^-channel inhibition contributes substantially to vinpocetine’s neuroprotective effects in vitro. Patch-clamp experiments showed that vinpocetine inhibits Na^+^ currents in cerebrocortical cultures (IC_50_ ≈ 44.2 µM) [58], prevents veratridine-induced cell death [87,88], and inhibits [^3^H]batrachotoxin binding in rat cortical synaptosomes (IC_50_ ≈ 0.34 µM) [57]. In synaptosomes, vinpocetine concentration dependently reduces veratridine-evoked Na^+^ accumulation and K^+^-depolarization–induced Ca^2+^ influx [61], suggesting concurrent Ca^2+^-channel inhibition. In hippocampal CA1 pyramidal neurons, vinpocetine suppresses veratridine-induced Ca^2+^ elevations and accelerates recovery to baseline levels, effects that likely contribute to neuroprotection [89]. Additional synaptosomal studies confirmed that low-micromolar vinpocetine suppresses Na^+^ and Ca^2+^ increases triggered by veratridine- or K^+^-depolarization, paralleling reduced glutamate and aspartate release—consistent with Na^+^-channel inhibition [90]. Vinpocetine also inhibits tetrodotoxin-sensitive Na^+^ channels (Na_v_1.8) in dorsal root ganglion–derived cells in a state- and concentration-dependent manner, producing a marked leftward shift in the inactivation curve without altering activation voltage dependence [91].

The significance of Na^+^-channel inhibition in vinpocetine’s mechanism of action has been highlighted by Adam-Vizi and colleagues [28,61,92] and by Sitges et al., who emphasized its relevance in suppressing neurotoxic glutamate release [72,93]. Although the original IC_50_ (44 µM) exceeds the ~1 µM concentrations associated with neuroprotective and anti-seizure effects, this discrepancy is explained by state-dependent affinity. Examination of Figure 2 in the original work shows that 40 µM vinpocetine reduces Na^+^-current amplitude to 71% at −70 mV, 36% at −60 mV, and 7% at −50 mV. Corresponding IC_50_ values (estimated via the Hill equation with 1:1 binding) are ~100 µM, ~23 µM, and ~3 µM, respectively. Thus, in depolarized, damaged neurons, vinpocetine may exert therapeutically relevant inhibition. Direct evidence of vinpocetine binding to Na^+^-channels comes from high-resolution cryo-electronmicroscopy structures of Na_v_1.7 showing clear ligand accommodation [94]. Because Na^+^-channel inhibitors preferentially silence hyperactive, damaged neurons, they generally do not impair global neural function, such as plasticity or memory. In a two-photon microscopy study examining dendritic spine motility—a proxy for synaptic plasticity—vinpocetine not only failed to reduce motility but also increased it, potentially contributing to its procognitive effects [95].

In 2024, the FDA approved suzetrigine, a selective Na_v_1.8 inhibitor, for the acute treatment of moderate-to-severe pain [96,97]. This discovery is expected to accelerate development of non-opioid NSAID analgesics, offering an alternative to drugs associated with significant societal and economic burdens.

Interestingly, vinpocetine inhibits both Na^+^ and Ca^2+^ elevations in synaptosomes exposed to the convulsant 4-AP, although the mechanism of the two effects differs. By blocking K^+^ permeability, 4-AP increases intracellular Na^+^, enhances transmitter release [98,99], and induces seizures. Sitges et al., therefore, concluded that vinpocetine possesses anticonvulsant activity [72,100], a conclusion supported by in vivo models such as pentilentetrazole-induced epilepsy [101]. Pilocarpine–lithium-induced seizures and associated histological, apoptotic, and neurotoxic changes were also significantly attenuated by subchronic vinpocetine pretreatment (14 days, 10 mg/kg, i.p.). Vinpocetine’s protection against epileptiform activity may involve inhibition of hippocampal PI3K/mTOR signaling [102]. The compound suppresses 4-AP–evoked epileptiform activity in human iPSC-derived neurons and reduces spontaneous and 4-AP–induced neuronal activity in zebrafish larvae without affecting locomotion [103]. Some authors therefore classify vinpocetine as a “third-generation” antiepileptic agent with favorable cognitive-enhancing properties [104], a view supported by human clinical observations [105].

GABA is the principal inhibitory neurotransmitter in the CNS, and vinpocetine’s anticonvulsant effects have also been linked to its actions on the GABAergic system. In a patient with Lennox–Gastaut syndrome carrying a GABRB3 Y302C (c.905A>G) mutation, six months of vinpocetine treatment (20 mg three times daily) markedly improved EEG activity and psychosomatic status. In HEK cells expressing wild-type GABRA3/GABRB3/GABRG2 receptors, vinpocetine was among the most potent GABA modulators identified in a screen of 1302 compounds (EC_50_ ≈ 291 nM) [106]. Another case report described significant improvement in seizure-free periods in a patient with autism spectrum disorder, psychiatric comorbidities, and therapy-resistant focal epilepsy carrying a loss-of-function GABRA1 mutation following vinpocetine treatment (40 mg for 16 months) [107].

### 5.3. Antioxidant Effects of Vinpocetine

Reactive oxygen species (ROS), including superoxide (O_2_•^−^), hydroxyl radicals (•OH), peroxyl radicals (ROO•), and alkoxyl radicals (RO•), are continuously generated during aerobic metabolism under physiological conditions. Endogenous antioxidant defenses—such as superoxide dismutase, catalase, glutathione peroxidase, glutathione reductase, reduced glutathione, and coenzyme Q—are responsible for neutralizing these reactive species. Oxidative stress arises when the equilibrium between ROS production and antioxidant capacity becomes disrupted, i.e., when antioxidant defenses are insufficient to eliminate the generated ROS. Excess ROS can attack lipids, proteins, and nucleic acids, leading to widespread structural and functional cellular damage. Because of its high content of polyunsaturated fatty acids and elevated oxygen consumption, the brain is particularly vulnerable to oxidative injury. Robust evidence indicates that oxidative stress contributes significantly to neuronal damage during cerebral ischemia and to the pathogenesis of neurodegenerative diseases such as Alzheimer’s disease, Parkinson’s disease, and multiple sclerosis. Glutamate release during ischemia (excitotoxicity), and especially subsequent reoxygenation, triggers pathways that generate highly toxic ROS, which play central roles in ischemia–hypoxia–induced cellular injury [108,109].

Under cell-free in vitro conditions, vinpocetine effectively inhibits hydroxyl radical (•OH) formation—species that are strongly cytotoxic and are produced during hypoxia–ischemia–reoxygenation [110]. Exposure of human erythrocytes to the superoxide-generating agent phenazine methosulfate markedly increases membrane rigidity and reduces filterability; vinpocetine (0.5–5 μM) significantly improved erythrocyte filterability, an effect attributed to its superoxide-scavenging activity [111]. In rat brain synaptosomes, oxidative-stress markers—including thiobarbituric acid reactive substances (TBARs), lipid peroxidation, and oxygen consumption—are strongly elevated in the presence of ascorbate/Fe^2+^ or 3-nitropropionic acid (3-NPA). Vinpocetine (25–100 µM) significantly inhibited these processes [112,113]. Miyamoto et al. demonstrated that antioxidants markedly attenuate glutamate-induced neurotoxicity in a neuronal cell line, and vinpocetine (10–100 µM) provided comparable protection, suggesting that its antioxidant properties contribute substantially to its cytoprotective effects [114]. In a randomised controlled clinical study, vinpocetine (10 mg/day for two weeks) significantly improved plasma and whole-blood viscosity, including erythrocyte rigidity indices, in patients with cerebrovascular disorders [115].

In vitro studies reveal complex, partly divergent effects of vinpocetine on mitochondrial function. Vinpocetine reduces H_2_O_2_ production in rat synaptosomes and guinea pig mitochondria, decreases Ca^2+^-induced Ca^2+^ release and mitochondrial swelling, and inhibits mitochondrial ATP synthesis while increasing ATPase activity. These multiple actions may involve distinct mitochondrial targets. Although inhibition of mitochondrial respiration may be viewed negatively, mild uncoupling and suppression of mitochondrial Na^+^/Ca^2+^ exchange may confer protective effects. Notably, inhibition of Ca^2+^-induced swelling and reduction of H_2_O_2_ production are considered unequivocally beneficial [116].

In primary hippocampal cultures, vinpocetine (0.5 µM) markedly protected against hypoxia (95% N_2_/5% CO_2_ for three h) followed by reoxygenation. Vinpocetine preserved mitochondrial membrane potential, reduced ROS production, and prevented antioxidants, including reduced glutathione [41]. β-Amyloid accumulation plays a central role in Alzheimer’s disease pathology. In PC12 cells, vinpocetine (40 µM) preserved cellular redox balance and counteracted oxidative injury induced by Aβ_1–40_ and Aβ_25–35_ peptides [117]. Intracerebral injection of ethidium bromide—a nucleic-acid binding fluorescent dye—induces demyelination and pronounced oxidative damage, including elevated malondialdehyde in cortex, hippocampus, and striatum, increased serum nitrite/oxide levels, and reduced glutathione. Vinpocetine (1.5 mg/kg) counteracted these changes in the striatum, whereas higher doses (3 or 6 mg/kg) produced slight exacerbation [118].

Lourenco-Gonzales et al. examined vinpocetine’s effects on pain and inflammation induced by the superoxide donor KO_2_. Vinpocetine (3–30 mg/kg ip.) dose-dependently attenuated KO_2_-induced pain, hyperalgesia, paw oedema, and leukocyte infiltration. Vinpocetine restored endogenous antioxidant status, reduced superoxide formation, and normalized NRF2 and HO) mRNA expression. It inhibited IκBα, thereby reducing NF-κB activation and the production IL-33, IL-1β, and TNF-α. The authors concluded that vinpocetine’s antioxidant activity underlies its inhibition of KO_2_-evoked pain and inflammation [119]. Protective effects of vinpocetine in lipopolysaccharide- or carrageenan-induced inflammation—including reduced cytokine release and suppression of NF-κB signaling—have similarly been attributed to its antioxidant properties. On this basis, vinpocetine has been proposed as a potential therapeutic agent for inflammatory pain [120,121].

### 5.4. Vinpocetine Inhibits Neuroinflammation

At the molecular level, the primary event triggered by cerebral ischemia is glutamate-mediated excitotoxicity, followed by secondary subacute processes. Among these, neuroinflammation represents a critical pathological process, distinct from peripheral inflammation. Neuroinflammation involves the activation of microglia and astrocytes after stroke or ischemia, accompanied by the release of proinflammatory mediators—including IL-1β, TNFα, and IL-6—which contribute to both acute neuronal injury and long-term neuronal remodeling [65,122,123,124,125].

Jeon et al. were the first to demonstrate that vinpocetine inhibits neuroinflammation, highlighting the central role of IκB kinase (IKK)–NF-κB pathway inhibition in this effect [23]. In the in vitro studies using vascular smooth muscle cells, endothelial cells, macrophages, and epithelial cells, they showed that vinpocetine suppresses TNFα-induced NF-κB activation and reduces the production of proinflammatory cytokines, including IL-1β, MCP-1, VCAM-1, ICAM-1 and MIP-2. Additionally, vinpocetine attenuated lipopolysacharide-induced lung inflammation through similar mechanisms. Mechanistically, vinpocetine directly inhibits IKK, preventing IκBα phosphorylation and degradation, independent of its phosphodiesterase inhibition or Ca^2+^-regulatory effects. The anti-inflammatory action of vinpocetine is further supported by its lack of activity on β-adrenergic receptors [62], whose stimulation typically suppresses proinflammatory cytokine production via cAMP elevation [126].

In animal models, vinpocetine modulates inflammation after cerebral ischemia. Following transient middle cerebral artery occlusion (MCAO) in mice, vinpocetine administration (5, 10, or 15 mg/kg, ip., for three days post-ischemia–reperfusion) reduced infarct volume and apoptosis in peri-infarct neurons. It increased Bcl-2 expression while suppressing Bax and cleaved caspase-3. Vinpocetine also inhibited microglial proliferation and NLRP3 inflammasome activation, resembling the effects of MCC950, a known NLRP3 inhibitor. These findings indicate that vinpocetine mitigates ischemia–reperfusion-induced inflammatory injury by inhibiting the NLRP3 inflammasome [127]. In a global ischemia model induced by four-vessel occlusion, vinpocetine (100 mg/kg, p.o., initiated five days post-occlusion for seven days) partially preserved hippocampal neurons and improved locomotor activity, showing a protective effect comparable to that of ozagrel, a thromboxane A2 synthesis inhibitor [128]. Similarly, in MCAO–reperfusion, vinpocetine (10 mg/kg, ip., 1 h post-ischemia) reduced infarct volume, cerebral oedema, and inflammatory markers, including NF-κB and TNFα expression [129].

Activation of NF-κB is a key driver of post-ischemic inflammation, and its inhibition by vinpocetine has been confirmed in human studies. In a clinical study of 60 patients with acute ischemic stroke, participants were randomized into a control group receiving standard therapy (*n* = 30) and a treatment group receiving vinpocetine (30 mg/day for 14 days) in addition to standard treatment. Peripheral blood mononuclear cells were analyzed at baseline, day 3, and day 7 for IκBα mRNA, total and phosphorylated IκBα, and proinflammatory mediators, including TNFα, IL-6, monocyte MCP-1, ICAM-1, and VCAM-1.

Plasma C-reactive protein (CRP) levels were also measured. Vinpocetine treatment significantly reduced inflammatory markers in mononuclear cells and plasma CRP, with detectable effects by day 7. Magnetic resonance spectroscopy revealed decreased MI/Cr ratios in peri-infarct regions, indicative of reduced inflammation. Although the study had limitations, it provides preliminary evidence that vinpocetine’s anti-inflammatory effects observed in animal models may also occur in humans, highlighting the need for larger clinical trials [130].

Toll-like receptors (TLRs) play a pivotal role in post-ischemic neuroinflammation. Wu et al. employed both MCAO and oxygen–glucose deprivation (OGD) models to investigate vinpocetine’s effects. Vinpocetine (10 mg/kg, ip.) reduced infarct volume, enhanced neuronal survival, decreased lactate dehydrogenase release, and attenuated apoptosis. Mechanistically, vinpocetine inhibited Toll-like receptor-4/myeloid-differentiation primary-response-88/nuclear-factor-kappa-light-chain-enhancer-of-activated-B-cells (TLR4/MyD88/NF-κB)–mediated proinflammatory cytokine production, neuronal degeneration, and apoptosis, suppressing TNFα and IL-1β release in both models [131]. Zhao et al. further confirmed vinpocetine’s anti-ischemic and antihypoxic effects in vitro and in vivo. In primary cortical astrocytes subjected to OGD/reoxygenation, vinpocetine preserved cell viability, reduced connexin 43 expression, ROS accumulation, NO release, and TNFα/IL-1β production. In rats subjected to MCAO–reperfusion, vinpocetine (10 mg/kg, ip., 30 min pre-MCAO) decreased infarct volume, cerebral edema, and oxidative stress (increased superoxide dismutase activity, decreased malondialdehyde). Vinpocetine restored p-Cx43 and p-AKT expression and p-Cx43/Cx43 and phospho-protein p-AKT/AKT) ratios, which were suppressed by ischemia–reperfusion. Co-administration of LY294002, a PI3K inhibitor, counteracted these effects, demonstrating that vinpocetine’s neuroprotection involves activation of the PI3K/AKT signaling pathway [132].

Exosomes—small extracellular vesicles released after ischemic injury—carry proteins, lipids, nucleic acids, and metabolites, influencing inflammation, degeneration, and intercellular communication [133]. Zang et al. reported that PDE1B expression is upregulated sevenfold in the peri-infarct region following MCAO. In microglial cultures subjected to OGD, vinpocetine (5–20 µM) modulated exosome biogenesis and uptake, significantly influencing neuronal survival. The authors proposed that PDE1B inhibition by vinpocetine alters microglial exosome release, enhancing autophagy, neuronal protection, and intercellular signaling [134].

Recently, it was reported that, in a model of cold-induced brain trauma in mice, a vinpocetine (5 or 10 mg/kg, ip., daily) dose-dependently reduced infarct volume, cerebral oedema, blood–brain barrier disruption, and motor cortex atrophy, while promoting ipsilateral neurogenesis and locomotor recovery. LC-MS/MS proteomic analysis identified 192 proteins whose expression was modulated by vinpocetine, highlighting potential novel therapeutic targets for further investigation [135].

#### Vinpocetine Reduces Microglia and Astroglia Activation

Microglia are a critical cell population in the central nervous system, responsible for recognizing and phagocytosing dead cells, maintaining brain homeostasis, producing neurotrophic factors, and releasing proinflammatory cytokines and extracellular vesicles. Microglial activation plays a pivotal role in brain inflammatory processes, neurodegenerative diseases, and both acute and chronic ischemic events [136].

The translocator protein (TSPO, 18 kDa) is a key marker of microglial activation [137]. Earlier, Gulyás et al. demonstrated that vinpocetine has significant affinity for TSPO, suggesting the potential use of [^11^C]vinpocetine in PET imaging to visualize microglial activation [59]. In BV-2 microglial cells, lipopolysaccharide (LPS) or oxygen-glucose deprivation (OGD) significantly increased TSPO expression, accompanied by enhanced cell damage and elevated production of IL-1β, IL-6, and TNFα. Vinpocetine (20–50 µM) significantly attenuated these effects. Similar protective effects were observed in the brains of mice exposed to hypoxia (8% O_2_, 95% N_2_), where vinpocetine (5 mg/kg, ip., daily for 7 days) prevented microglial activation and cytokine overproduction [138].

In astrocyte cultures, vinpocetine (0.1 µM) reduced hypoxia-induced cell death and apoptosis, preserved mitochondrial function, and enhanced ATP and phosphocreatine levels before apoptosis. Additionally, astrocyte proliferation during reoxygenation was increased [139]. In rat primary astrocyte cultures, OGD/reoxygenation induced injury characterized by elevated LDH activity, apoptosis, reactive oxygen species (ROS) production, decreased superoxide dismutase (SOD) activity, and increased MDA levels. Vinpocetine treatment attenuated all these parameters. Parallel in vivo studies using MCAO confirmed these findings: ischemia/reperfusion triggered oxidative stress, inflammatory responses, and apoptotic pathways Fas, TNFα, and TRAIL receptor-mediated caspase-3 activation), which were mitigated by vinpocetine. The protective effect is partly attributed to phosphorylation of Cx43 via activation of the PI3K/AKT pathway [132].

### 5.5. Vinpocetine Inhibits Ca^2+^/Calmodulin-Dependent PDE1 Enzymes

Phosphodiesterases (PDEs) are a diverse superfamily of enzymes responsible for hydrolyzing cyclic nucleotides cyclic adenosine monophosphate (cAMP) and cyclic guanosine monophosphate (cGMP) thereby regulating intracellular signaling. PDEs are divided into 11 families (PDE1–PDE11), with distinct substrate specificity and tissue distribution. PDE1, PDE2, PDE3, PDE10, and PDE11 hydrolyze both cAMP and cGMP, while PDE1 is unique in its Ca^2+^/calmodulin (CaM)-dependent activation. PDE1 has three subtypes (PDE1A, PDE1B, PDE1C) with distinct regional expression in the brain: PDE1A is abundant in the hippocampus and cerebral cortex, PDE1B in dopaminergic regions such as the striatum and nucleus accumbens, and PDE1C in the cerebellum and olfactory epithelium. Notably, PDE1B expression in the striatum and nucleus accumbens is 2–10 times higher than in other regions or peripheral tissues. By inhibiting PDE1, intracellular levels of cAMP and cGMP increase, activating downstream PKA and PKG and modulating multiple processes, including vascular tone, memory, cognition, inflammation, synaptogenesis, mitochondrial biogenesis, ATP synthesis, antioxidant enzyme expression, and immune responses [14,17,55,140,141,142,143,144,145,146,147,148,149]. PDE inhibitors have been explored for treating neurodegenerative diseases such as Alzheimer’s disease and mild cognitive impairment, although clinical efficacy has been limited [9,16,150,151].

Vinpocetine was shown to inhibit both basal and Ca^2+^/CaM-activated PDE1 activity in rat and bovine brain tissues, with IC_50_ values ranging from 14–200 µM depending on substrate and experimental conditions [23,48,51,52,53,54,55,56]. Vinpocetine preferentially inhibits PDE1A and PDE1B (IC_50_ ~10 µM) over PDE1C (IC_50_ ~40 µM). Its vasorelaxant effects are attributed primarily to PDE1 inhibition, leading to elevated cGMP and reduced Ca^2+^-induced cytosolic calcium release [53].

In vivo, vinpocetine-mediated PDE1 inhibition contributes to multiple neuroprotective effects. For example, in rodent models of fetal alcohol spectrum disorder, vinpocetine (10–20 mg/kg, ip.) alleviated cognitive deficits and restored ocular dominance plasticity via cAMP/cGMP-mediated signaling [152,153]. Developmental exposure to lead induced hyperactivity, impaired orientation, decreased hippocampal cAMP levels, and abnormal CREB phosphorylation, all of which were significantly reversed by vinpocetine treatment. Similarly, hyperserotonemia-induced behavioral and biochemical alterations such as BDNF), pCREB), (IL-6), interleukin-10 (IL-10), TNFα, TBARs, GSH in rodent offspring were corrected by vinpocetine (10–20 mg/kg, s.c.) [154,155,156,157]. ADHD-like symptoms in prenatal alcohol-exposed rats were also moderated by vinpocetine [158].

Vinpocetine has additional PDE1-dependent antioxidant and neuroprotective effects. Combined with moderate exercise, vinpocetine (10 mg/kg/day, ip., 3 weeks) improved systemic redox status by reducing ROS and increasing antioxidant enzyme activity [159]. In models of β-amyloid-induced cognitive impairment, vinpocetine (4 mg/kg, p.o., 30 days) improved memory and learning, and restored hippocampal long-term potentiation and synaptic plasticity [160,161]. Huntington’s disease-like symptoms induced by 3-nitropropionic acid were mitigated by vinpocetine, improving oxidative stress and mitochondrial function [162]. PDE1 inhibition may also contribute to vinpocetine’s ability to improve red blood cell deformability, as demonstrated in sickle cell anemia models [163,164].

### 5.6. Vinpocetine and the Purinergic System

Adenosine is a key inhibitory neuromodulator in the central nervous system, with physiological extracellular concentrations ranging from 20 to 300 nM [165]. During cerebral hypoxia or ischemia, neuronal depolarization triggers the release of large amounts of ATP into synapses, which is rapidly converted into adenosine by ectonucleotidases, raising extracellular adenosine to approximately 30 µM—roughly 100-fold above resting levels [166,167]. Adenosine exerts concentration-dependent effects: at low levels, it provides neuroprotection via A1 receptor activation [168], whereas at high concentrations it activates microglial and astrocytic A2B receptors, promoting proinflammatory cytokine production and contributing to neuronal injury [167].

The interaction of vinpocetine with the purinergic system is incompletely understood. Vinpocetine inhibits veratridine-induced purine release in rat hypothalamic synaptosomes and reduces adenosine uptake into red blood cells. Its anti-anoxic effects are partly attributed to the suppression of hypoxia-induced adenosine release, thereby limiting its metabolism to xanthine and hypoxanthine, which generate free radicals upon reoxygenation [169]. Milusheva et al. showed that hypoxia reduces [^3^H]acetylcholine release from isolated Auerbach plexus smooth muscle, an effect partially reversed by vinpocetine (100 µM) independently of adenosine receptor interaction. Vinpocetine did not influence presynaptic adenosine action, but, when combined with the P1 receptor antagonist theophylline, it fully reversed hypoxia-induced effects, suggesting that vinpocetine acts primarily by modulating Ca^2+^ homeostasis rather than by direct adenosine receptor activity [170]. Similarly, Krieglstein et al. demonstrated that vinpocetine alone did not protect against cyanide-induced cytotoxic hypoxia in chicken embryonic cells, but potentiated the protective effects of adenosine, particularly at low concentrations (0.1–10 µM). They hypothesized that vinpocetine’s inhibition of neuronal adenosine (re)uptake activates postsynaptic A1 receptors, leading to neuronal hyperpolarization and reduced Ca^2+^ influx [171].

## 6. Summary—Mechanism of Action of Vinpocetine

**Figure 3:**
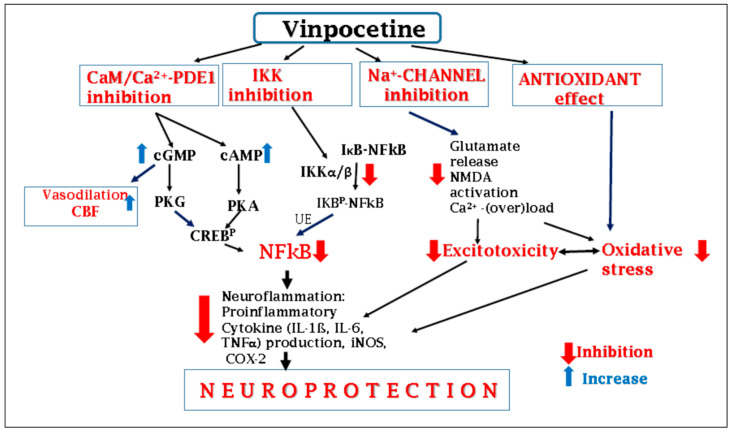
Mechanism of action of vinpocetine in brain ischemia. Vinpocetine exerts neuroprotective effects through multiple, complementary mechanisms that converge on key pathways involved in ischemic and hypoxic brain injury. Phosphodiesterase-1 (PDE1) Inhibition: Vinpocetine inhibits Ca^2+^/calmodulin-dependent PDE1 enzymes, leading to increased intracellular levels of cyclic nucleotides, cAMP and cGMP. Elevated cAMP/cGMP activates protein kinase A (PKA) and protein kinase B (PKB), which, via downstream intermediates such as CREB activation, suppress the IKK/NF-κB signaling pathway. This results in reduced expression of proinflammatory mediators, including TNF-α, IL-1β, IL-6, inducible nitric oxide synthase (iNOS), and cyclooxygenase-2 (COX-2). IKK Inhibition: vinpocetine directly inhibits IκB kinase, preventing the phosphorylation and degradation of IκBα, thereby blocking NF-κB activation. This mechanism directly suppresses the production of inflammatory mediators, including TNF-α, IL-1β, IL-6, iNOS, and COX-2. Sodium channel blockade and excitotoxicity reduction: vinpocetine inhibits voltage-gated sodium channels, particularly Na_v_1.8, and counteracts glutamate-mediated excitotoxicity. By preventing excessive activation of NMDA receptors, it reduces intracellular Ca^2+^ overload, Na^+^ influx, and the subsequent activation of Ca^2+^-dependent kinases. This cascade attenuates IKK/NF-κB activation and limits the production of proinflammatory mediators. Antioxidant effects: Vinpocetine scavenges reactive oxygen species generated during ischemia and ischemia-reperfusion, preventing oxidative stress-induced activation of the IKK/NF-κB pathway. Consequently, the production of inflammatory mediators such as TNF-α, IL-1β, IL-6, iNOS, and COX-2 is reduced. Current drug discovery approaches rely on well-characterized molecular targets, but investigation of compounds like vinpocetine can reveal novel targets or molecular networks, potentially guiding the development of more effective derivatives. Considering recent insights into ischemic brain pathology, as well as vinpocetine’s multimodal actions—i.e., antioxidant effects, phosphodiesterase (PDE) and sodium channel inhibition, and suppression of neuroinflammation—demonstrated by in vitro and in vivo experimental data, these findings support its use in the treatment of ischemic–hypoxic neural injury.

Vinpocetine is a multi-target compound with a complex mechanism of action (Figure 3). Preclinical studies demonstrate that its neuroprotective effects in cerebral ischemia involve:

• Phosphodiesterase-1 inhibition, enhancing intracellular cAMP and cGMP signaling.
• Voltage-gated sodium channel blockade, reducing excitotoxicity.
• Antioxidant activity, attenuating oxidative stress.
• Anti-inflammatory effects, primarily through inhibition of the IKK/NF-κB pathway, reducing the production of proinflammatory mediators in neuroinflammatory processes.
• In various experimental in vitro systems in which the major individual effects of vinpocetine are investigated separately with respect to specific alterations, the compound’s action can generally be demonstrated at low micromolar concentrations. However, it is likely to be hypothesized that, under in vivo conditions (e.g., in cerebral ischemia and hypoxia), if the compound is present, its individual effects may act in an additive or synergistic manner; thus, its protective effect (e.g., against ischemic damage) may also be evident at lower concentrations.

## 7. Potential Clinical Applications

Based primarily on preclinical findings (see Section 5), as well as numerous clinical pilot studies and network meta-analyses, vinpocetine is recognized as a neuroprotective agent with a complex and multifaceted mechanism of action. Its pharmacological effects include inhibition of phosphodiesterase type 1 (PDE1), blockade of voltage-gated sodium channel subtype Nav1.8, attenuation of oxidative stress, and suppression of neuroinflammatory processes triggered by cerebral ischemia and hypoxia.

Owing to its favorable safety profile and the absence of significant adverse effects or toxicity, vinpocetine has attracted considerable interest from both preclinical and clinical researchers, as well as from the pharmaceutical industry, in the search for novel therapeutic indications. Evidence suggests that vinpocetine is safe for long-term administration at therapeutic doses [172], which has further stimulated investigations into its additional therapeutic applications, molecular mechanisms, and pharmacological targets. Due to these properties, Vinpocetine has also become widely available worldwide as a dietary supplement.

Current treatment options for acute ischemic stroke remain largely confined to reperfusion strategies, including intravenous thrombolysis and endovascular thrombectomy [173]. As a neuroprotective agent, vinpocetine has been clinically utilized in several countries to improve neurological function and prognosis in patients with cerebrovascular disorders, including stroke [5,7,130,174]. Clinical studies have also reported beneficial effects in patients with senile dementia and memory impairment [16,151,175].

In clinical practice, several neuroprotective agents—such as citicoline [176], cerebrolysin [177,178], minocycline [179], and vinpocetine [7]—have been used as adjunctive therapies. These agents have demonstrated varying degrees of efficacy in improving neurological outcomes and functional prognosis in patients with acute ischemic stroke. Collectively, these findings underscore the potential clinical value of neuroprotective strategies, including vinpocetine, in the management of acute ischemic stroke.

Furthermore, transcranial Doppler studies have provided evidence that vinpocetine enhances cerebral blood flow and oxygenation in patients with stroke [43]. Improvements in neurological function have also been reported [172], reinforcing earlier preclinical and clinical observations suggesting beneficial effects on memory performance and cognitive function.

To further establish the clinical efficacy of neuroprotective agents, including vinpocetine, large-scale, multicenter randomized controlled trials are warranted [5,151,172].

## 8. Conclusions

Since its clinical introduction nearly 50 years ago, interest among scientists and the pharmaceutical industry in vinpocetine has increased significantly, largely due to its beneficial multifaceted biochemical actions associated with cerebral hypoxic injury, and very low incidence of side effects. Based on reliable neurochemical and pharmacological data, namely its sodium channel type 1.8 and phosphodiesterase 1-blocking properties and proven antioxidant and anti-inflammatory effects, vinpocetine, as a neuroprotective agent, is justifiably used in the treatment of post-stroke and other cerebral ischemic conditions. In addition, its cerebrovascular-enhancing properties, observed in animal and clinical studies, are linked to improvements in patients with memory problems.

## Figures and Tables

**Table 1:** Major reviews about vinpocetine.

	(2000–2025)	
Author	Title	Journal
Abu Alghayt, MH. et al. [1]	Atheroprotective role of vinpocetine: an old drug with new indication.	Inflammopharmacology. 2024; 32:3669–3678, doi.org/10.1007/s10787-024-01529-5
Alshehri, A. et al. [2]	The anti-inflammatory properties of vinpocetine mediates its therapeutic potential in management of atherosclerosis.	J Inflammation. 2024; 21:1–19. doi.org/10.1186/s12950-024-00394-x
Al-Kuraishy, HM. et al. [3]	New insights on the potential effect of vinpocetine in Parkinson’s disease: one of the neglected warden and baffling topics.	Metabolic Brain Disease. 2023; 38:1831–1840. doi.org/10.1007/s11011-023-01254-y
Puig, N. et al. [4]	Novel therapeutic approaches to prevent atherothrombotic ischemic stroke in patients with carotid atherosclerosis.	Int J Mol Sci; 2023; 24(14325):1–16. doi.org/10.3390/ijms241814325
Li, Y. et al. [5]	The efficacy and safety of post-stroke cognitive impairment therapies: an umbrella review.	Front Pharmacol. 2023; 14(1207075). doi. 10.3389/fphar.2023.1207075
Gan, J. et al. [6]	Anti-inflammatory therapy of atherosclerosis: focusing on IKKß.	Journal of Inflammation. 2023; 20, doi.org/10.1186/s12950-023-00330-5
Panda, PK. et al. [7]	Safety and efficacy of vinpocetine as a neuroprotective agent in acute ischemic stroke: a systematic review and meta-analysis.	Neurocrit Care. 2022; 37:314–325. doi.org/10.1007/s12028-022-01499-y
Balaha, M. et al. [8]	Vinpocetine’s immunomodulating, anti-oxidant, anti-inflammatory, anti-fibrotic, and PDE inhibiting potencies ameliorate bleomycin- induced pulmonary fibrosis.	Iran J Basic Med Res. 2023; 26:13–22. doi: 10.22038/IJBMS.2022.64175.14130.
Sheng, J. et al. [9]	Inhibition of phosphodiesterase: A novel therapeutic target for the treatment of mild cognitive impairment and Alzheimer’s disease.	Front Aging Neurosci. 2022; 14:1019187. 10.3389/fnagi.2022.1019187
Al-Kuraishy, HM. et al. [10]	Role of vinpocetine in ischemic stroke and poststroke outcomes: A critical review.	Brain Circulation. 2020; 6:1–10. doi: 10.4103/bc.bc_46_19
Zhang, C. et al. [11]	Updates of recent vinpocetine research in treating cardiovascular diseases.	J Cell Immunol, 2020; 211–219 doi: 1033696/immunology.2.045.
Dubey, A. et al. [12]	Review on vinpocetine.	Int J Pharm Life Sci. 2020; 11:6590–6597 www.reserchgate.net/publication/344467105
Zuo, H. et al. [13]	Phosphodiesterases as therapeutic targets for respiratory diseases.	Pharmacol Ther. 2019; 197:225–242 doi.org/10106/j.pharmthera.2019.02.002
Wu, Y. et al. [14]	Novel phosphodiesterase inhibitorsfor cognitive improvement in Alzheimer’s disease.	J Med Chem. 2018; 61:5467–5473. doi.org/10.1021/acs.jmedchem.7b01370
Zhang, Y-S. et al. [15]	An update on vinpocetine: New discoveries and clinical implications.	Eur J Pharmacol. 2018; 819:30–34, doi.org/10.1016/j.ejphar.2017.11.041
Prickaerts, J. et al. [16]	Investigational phosphodiesterase inhibitors in phase I and phase II clinical trials for Alzheimer’s disease.	Exp Opin Investig Drugs. 2017; 26:1033–1048, doi.org/10.1080/13543784.2017.1364360
Knott R, et al. [17]	Phosphodiesterase inhibitors as a therapeutic approach to neuroprotection and repair.	Int J Mol Sci. 2017; 18(696). doi:10.3390/ijms18040696
Yan, OC. et al. [18]	Cyclic nucleotide phospho-diesterase 1 and vascular aging.	Clinical Science 2015; 129:1077–1081 doi: 10.1042/CS20150605
Zhang, L. et al. [19]	Anti-Inflammatory effects of vinpocetine in atherosclerosis and ischemic stroke: a review of the literature.	Molecules. 2015; 20:335–347. doi:10.3390/molecules20010335
Patyar, S. et al. [20]	Role of vinpocetine in cerebrovascular diseases.	Pharmacological Report. 2011; 63(3):618–628.
Sitges, M. [21]	Antiepileptic drugs targeting cerebral presynaptic ion channels reduce cerebral excitability decreasing glutamate release.	In: Novel Treatment of Epilepsy 2011; 111–132. ed. Humberto Foyaca-Sibat, InTech,
Medina, AE. [22]	Vinpocetine as a potent anti-inflammatory agent.	PNAS. 2010; 107(22):9921–9922, doi/10.1073/pnas.1005138107
Jeon, K-I. et al. [23]	Vinpocetine inhibits NF-kB-dependent inflammation via an IKK-dependent but PDE-independent mechanism.	PNAS 107(21):9795–9800, 2010. doi/10.1073/pnas.0914414107
Bereczki, D. et al. [24]	Vinpocetine for acute ischemic stroke.	Cohrane Database Syst Rev.2008 (Jan 23 (1): CD00480):2404–2405, 2008. doi: 10.1002/14651858.CD000480.pub2.
Szatmári, S. et al. [25]	Vinpocetine for cognitive impairment and Dementia.	Cochrane Database of Systematic Reviews 2003, Issue 1. Art. No.: CD003119. doi: 10.1002/14651858.CD003119
Vas, Á. et al. [26]	Clinical and non-clinical investigations using positron emission tomography, near infrared spectroscopy and transcranial Doppler methods on the neuroprotective drug vinpocetine: a summary of evidences.	J Neurol Sci. 2002; 203–204:259–262, doi: 10.1016/s0022-510x(02)00301-5.
Dézsi, L. et al. [27]	A vinpocetin neuroprotektív hatásai in vivo és in vitro.	Acta Pharmaceutica Hungarica 2002; 72:84–91
Bönöczk, P. et al. [28]	Role of sodium channel inhibition in neuroprotection: Effect of vinpocetine.	Brain Res. Bull. 2000; 53:245–254. doi: 10.1016/s0361-9230(00)00354-3

**Table 2:** Identified molecular targets of vinpocetine.

Enzyme/Channel/ Receptor	Tissue/Cell	IC_50_ (µM)	Effect	References
Ca^2+^/Calmodulin PDE	rat, bovine aorta	14–200	cAMP, cGMP elevation	[23,48,49,51,52,53,54,55,56]
voltage-dependent Na^+^-channels	primary cell culture of rat brain	44.7	Na^+^-channel block	[57]
	rat cortical neuron	44.2	blockade of Na^+^-currents	[58]
[^3^H]-Batrachotoxin binding	rat cerebrocortical synaptosomes	0.34	Na_V_-channel binding	[57]
	rat brain	1.9	Na^+^-channel	[59]
Ca^2+^ channel	*Xenopus* oocytes	~100	inhibition of voltage-dependent Ca^2+^-channels	[60]
	isolated nerve terminals	1–20	inhibition of Ca^2+^-induced depolarization	[61]
Ca^2+^-channel, L-type	rat cerebral cortex	2.1	inhibition of Ca^2+^-induced depolarization	[59]
NFκB dependent transcriptional activity	vessel smooth muscle	25	inhibition of neuro- inflammation	[23]
IKK	vessel smooth muscle cells	26	inhibition of neuro- inflammation	[23]
Translocator protein (TSPO)	rat heart	0.2	steroidogenesis, apoptosis, oxidative stress	[59]

## Data Availability

Not applicable, No new data were created or analyzed in this study.

## Abbreviations

4-AP	4-aminopyridine
AMPA	α-amino-3-hydroxy-5-methyl-4-isoxazolepropionic acid
Bax	Bcl-2-associated X protein
Bcl-2	B-cell lymphoma
BDNF	brain-derived neurotrophic factor
CBF	cerebral blood flow
CNS	central nervous system
COX-2	cyclooxygenase 2
Fas	cell surface receptor (called CD91 or APO-1)
GABA	γ-Aminobutyric acid
GSH	reduced glutathione
HO-1	heme oxygenase-1
ICAM-1	intercellular adhesion molecule-1
IKK	IκB kinase
IL-10	interleukin-10
IL-1β	interleukin-1β
IL-33	interleukin 33
IL-6	interleukin-6
iNOS	inducible nitrogen oxide synthase
iPSC	induced pluripotent stem cell
IκBα	inhibitor of κB alpha degradation
LDH	lactate dehydrogenase
MCP-1	monocyte chemoattractant protein 1
MDA	malondialdehyde
MIP-2	macrophage inflammatory protein
NF-κB	nuclear factor kappa-light-chain-enhancer of activated B cells
NLRP+	NOD-like receptor family, pyrin domain containing
NMDA	N-methyl-D-aspartate
NRF2	Nuclear factor erythroid 2 related factor
NSAID	non-steroidal anti-inflammatory drug
p-AKT	phospho-protein kinase B
p-AKT/AKT	p-Cx43/Cx43 and phospho-protein kinase B/protein kinase B
pCREB	phosphorylated cAMP response element-binding protein
p-Cx43	connexin 43
PI3K	phosphatidylinositol kinase
PI3K/mTOR	phosphatidylinositol kinase/mechanistic Target Of Rapamycin
PKA	cAMP dependent protein kinase A
PKB	cGMP dependent protein kinase
TBARs	thiobarbituric reactive substances
TNFα	tumor necrosis alpha
TRAIL	tumor necrosis factor–related apoptosis-inducing ligand.
UE	ubiquitin enzyme
VCAM-1	vascular cell adhesion protein 1
